# Biomechanical and clinical evaluation of the posterosuperior screw in-out-in in the inverted triangular cannulated screws fixation of femoral neck fractures

**DOI:** 10.3389/fbioe.2026.1914058

**Published:** 2026-09-23

**Authors:** Jian Zhang, Jihai Xu, Huan Zou, Yaopeng Huang, Xin Tang, Hong Chen, Xin Wang

**Affiliations:** 1 Department of Plastic Reconstructive Surgery & Hand Microsurgery, Ningbo No.6 Hospital, Ningbo, China; 2 Ningbo Clinical Research Center for Orthopedics, Sports Medicine & Rehabilitation, Ningbo, China; 3 Department of Orthopaedic Trauma, First Affiliated Hospital of Dalian Medical University, Dalian, China

**Keywords:** femoral neck fracture, cannulated screw, in-out-in, biomechanics, finite element analysis, avascular necrosis

## Abstract

**Purpose:**

The aim of this study is to evaluate the mechanical performance and clinical outcomes of the posterosuperior screw In-Out-In (IOI) and All-In in the inverted triangular cannulated screws fixation of femoral neck fractures.

**Methods:**

Comparison of the mechanical properties of three inverted triangular cannulated screws fixation configurations, IOI, All In-Low (AI-L), and All In-Narrow (AI-N), for femoral neck fractures with different Pauwels angles using finite element analysis. Biomechanical experiments involving torsion, anteroposterior compression, axial compression, and cyclic loading were conducted using ORTHObones artificial femurs. We retrospectively analyzed postoperative data from 91 patients with femoral neck fractures. Based on hip CT scans, patients were divided into the IOI group (32 cases) and the All-In group (59 cases), and the incidence of nonunion and avascular necrosis (AVN) of the femoral head were compared between the two groups.

**Results:**

Finite element and biomechanical experiments demonstrated that the IOI group exhibited superior performance compared to the AI-L and AI-N groups in terms of torsional, bending, shear, and fatigue resistance. The clinical follow-up revealed that the incidence of nonunion in the IOI and All-In groups was 9.38% and 8.47%, respectively, and the incidence of AVN was 15.63% and 15.25%, with no statistically significant differences between the groups (*P* > 0.05).

**Conclusion:**

Our study demonstrated that the IOI configuration exhibited superior mechanical performance compared to the All-In configuration, while not increasing the risk of nonunion or AVN, and thus may not justify repeated fluoroscopy to avoid this trajectory. However, given the retrospective nature and limited sample size, these conclusions require confirmation through larger prospective studies with longer follow-up.

## 1 Introduction

Femoral neck fractures are common in clinical practice, accounting for approximately 53% of all hip fractures (Lin et al., 2024). Cannulated screw fixation remains the preferred approach for hip-preserving treatment of femoral neck fractures (De Franco et al., 2023). Conventionally, three screws are inserted in parallel in an inverted triangular configuration, placed within 3 mm of the femoral neck cortex to ensure stable support (Wang et al., 2022). However, owing to the unique anatomical characteristics of the femoral neck cross-section, which resembles an oblique ellipse with the relatively greater bone deficiencies in the posterosuperior and anteroinferior regions (Deng et al., 2023), the posterosuperior screw frequently penetrates the femoral cortex anatomically, even when such penetration is not apparent fluoroscopically. This phenomenon, referred to as In-Out-In (IOI), occurs at a rate ranging from 54% to 70% (Hoffmann et al., 2019; Yuan et al., 2020).

Regarding the clinical implications, previous studies have suggested that the IOI posterosuperior screw penetrates the femoral neck adjacent to the superior retinacular artery, which is situated at the 11 o’clock position on the femoral neck cross-section (when viewed along its long axis from distal to proximal) (Lavigne et al., 2005; Rego et al., 2017). Due to the critical role of the superior retinacular artery for femoral head perfusion, it has been recommended to avoid the posterosuperior screw IOI to minimize the potential risk of arterial injury and thereby reduce the incidence of avascular necrosis (AVN) of the femoral head. For instance, Kuttner et al. placed the posterosuperior screw below the piriformis fossa under anteroposterior (AP) fluoroscopy to ensure that the screw remained within the femoral neck (All In-Low, AI-L) (Kuttner et al., 2023). Zhang et al. inserted the posterosuperior screw anteriorly under lateral fluoroscopy, creating a narrow inverted triangular screw configuration to avoid the posterosuperior screw IOI (All In-Narrow, AI-N) (Zhang et al., 2023). However, it is worth noting that no anatomical or radiological studies have visually demonstrated the risk of the superior retinacular artery injury associated with the IOI posterosuperior screw. Furthermore, no clinical studies have focused on the impact of the IOI posterosuperior screw on AVN following femoral neck fractures.

According to the AO principles of fracture management (Helfet et al., 2003), fracture fixation is always a compromise that balances biology and biomechanics. A stable biomechanical environment promotes femoral head revascularization and trabecular reconstruction following femoral neck fracture (Xiong et al., 2019). Nevertheless, the biomechanical effect of the IOI posterosuperior screw on femoral neck fractures remains controversial. Yuan et al. suggested that the IOI posterosuperior screw may compromise the integrity of the femoral neck cortex, potentially reducing overall stability (Yuan et al., 2020). Conversely, some studies have proposed that the IOI screws provide rigid stability through multiple cortical purchases (Sen et al., 2012). To the best of our knowledge, the current literature lacks biomechanical experimental studies evaluating the influence of the IOI posterosuperior screw on the stability of femoral neck fractures.

In summary, to address the existing knowledge gap concerning the influence of the IOI posterosuperior screw on femoral neck fractures, our study aimed to evaluate its impact on the mechanical stability of femoral neck fractures using finite element analysis (FEA) and biomechanical experiments. Additionally, we assessed its effect on the prognosis of femoral neck fractures (including AVN and nonunion) through clinical studies.

## 2 Methods and materials

Our study was approved by the Ethics Committee of the First Affiliated Hospital of Dalian Medical University (number PJ-KS-KY-2024–250) and performed in accordance with the Helsinki Declaration.

### 2.1 FEA

We collected femoral computed tomography (CT) images from a 29-year-old male volunteer and imported them into Mimics 21.0 (Materialise, Belgium) to construct the three-dimensional femoral model. Geomagic Studio 2021 (Raindrop Inc., USA) was used to generate the non-uniform rational B-spline (NURBS)-wrapped surfaces, which were subsequently imported into SolidWorks (Dassault, France) (Liu et al., 2024). To simulate 30° Pauwels type I, 50° Pauwels type II and 70° Pauwels type III femoral neck fractures, osteotomies of 20°, 40°, and 60° were performed at the center of the femoral neck relative to the femoral neck axis according to previous literature (Shen et al., 2016) (Figure 1A).

**FIGURE 1:**
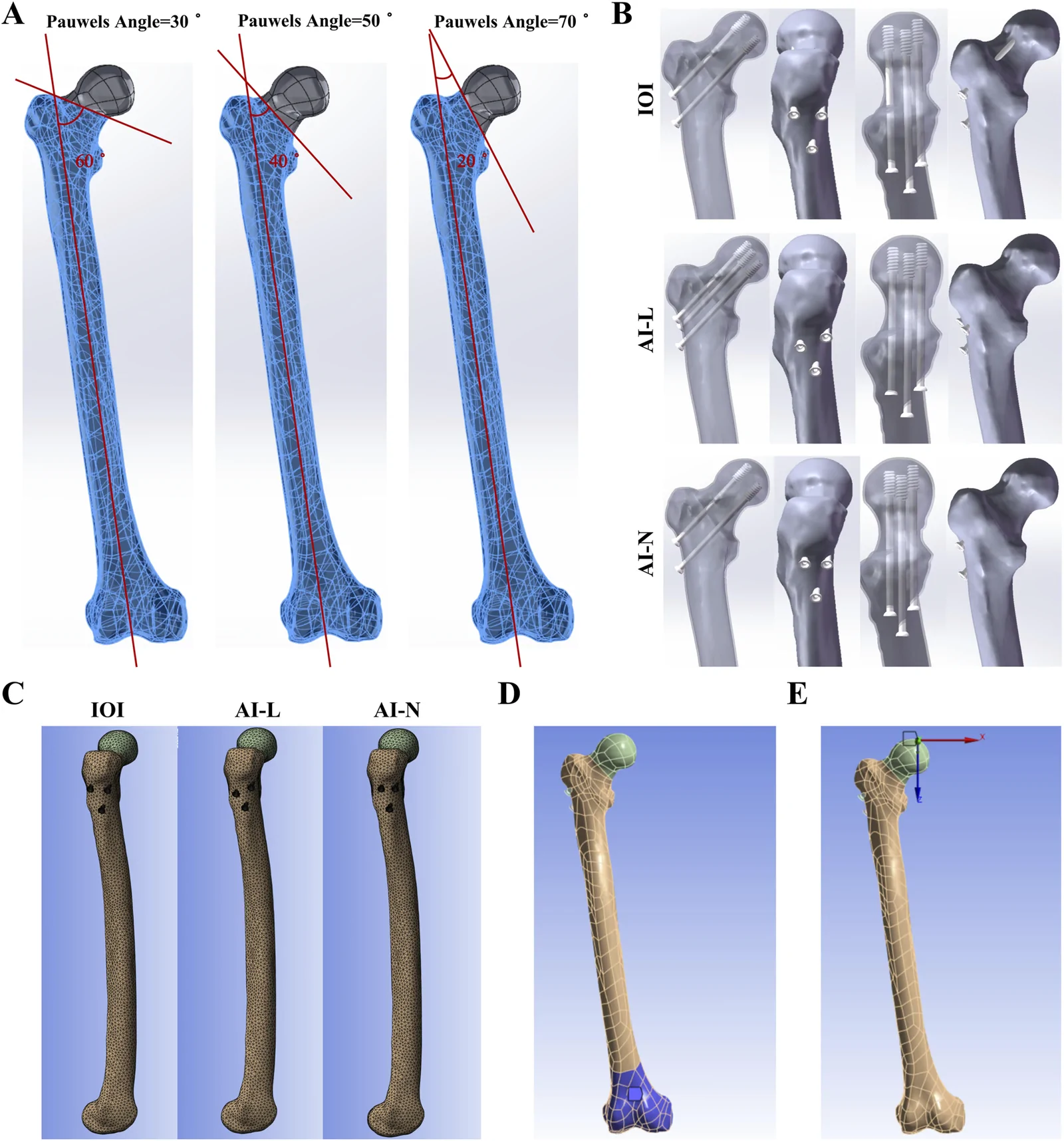
Finite element modeling and boundary conditions. **(A)** 30° Pauwels type I, 50° Pauwels type II and 70° Pauwels type III femoral neck fractures models, with osteotomy angles of 20°, 40°, and 60° relative to the femoral neck axis, respectively. **(B)** Three inverted triangle screw configurations: IOI, AI-L, and AI-N. The positions of the inferior screw and the anterosuperior screw are identical across all configurations, with differences only in the posterosuperior screw positions. **(C)** The hybrid mesh strategy improves the computational accuracy of femoral neck modeling by enhancing resolution through an increase in the number of nodes and elements. **(D)** Boundary constraints of the distal femur with 10° abduction and 9° posterior inclination to simulate single-leg stance. **(E)** Apply a load of 2100 N (equivalent to 300% of body weight) to the weight-bearing area of the femoral head.

Based on standard clinical parameters for cannulated screws, the screw thread diameter was defined as 7.3 mm, the thread length at 16 mm, and the shaft diameter at 4.8 mm. We created three configurations of inverted triangular screws, designated as the posterosuperior screw IOI, AI-L, and AI-N. The positions of the inferior screw and the anterosuperior screw in these three groups were positioned identically adjacent to the femoral neck cortex, with the only difference being the position of the posterosuperior screw (Figure 1B). The three configurations of inverted triangular screws were virtually inserted into the three Pauwels type femoral neck fractures.

Subsequently, the models were imported into Ansys Workbench 14.5 (ANSYS, Inc., USA) for simulation. The femur and cannulated screw modeled in this study were considered continuous, isotropic, and homogeneous linear elastic materials. The elastic modulus and Poisson’s ratio of cortical bone were 16350 MPa and 0.26, respectively. Those of cancellous bone are 137 MPa and 0.3, respectively. The cannulated screw was made of titanium alloy (Ti-6Al-7Nb), with the elastic modulus of 110000 MPa and the Poisson’s ratio of 0.3 (Wang et al., 2026). Based on previous studies, bonded contact was applied between the screw and the femur, while friction contact with a coefficient of 0.46 was used at the fracture surface (Long et al., 2024). Our study employed a hybrid mesh strategy to improve the computational accuracy of femoral neck modeling by increasing the number of nodes and elements to enhance resolution (Figure 1C).

The boundaries of the distal femur were constrained, with 10° abduction and 9° inclined posteriorly, to simulate the single-leg standing position (Figure 1D) (Zhang et al., 2022). A force of 2100 N corresponding to 300% of the body weight was applied at the weight-bearing region of the femoral head (Figure 1E). The peak von Mises stress and displacement of the femur and cannulated screws were measured in each group.

### 2.2 Biomechanical analysis

ORTHObones artificial femurs (3 B Scientific, Germany) were randomly divided into three groups: the IOI group, the AI-L group, and the AI-N group, corresponding to the finite element groups, with 15 femurs in each group (Figures 2A–C) (Kim et al., 2022). Each group was randomly divided into three subgroups, and osteotomy procedures were performed using custom-made osteotomy jig to simulate 30° Pauwels type I, 50° Pauwels type II and 70° Pauwels type III femoral neck fractures, respectively (Figure 3A).

**FIGURE 2:**
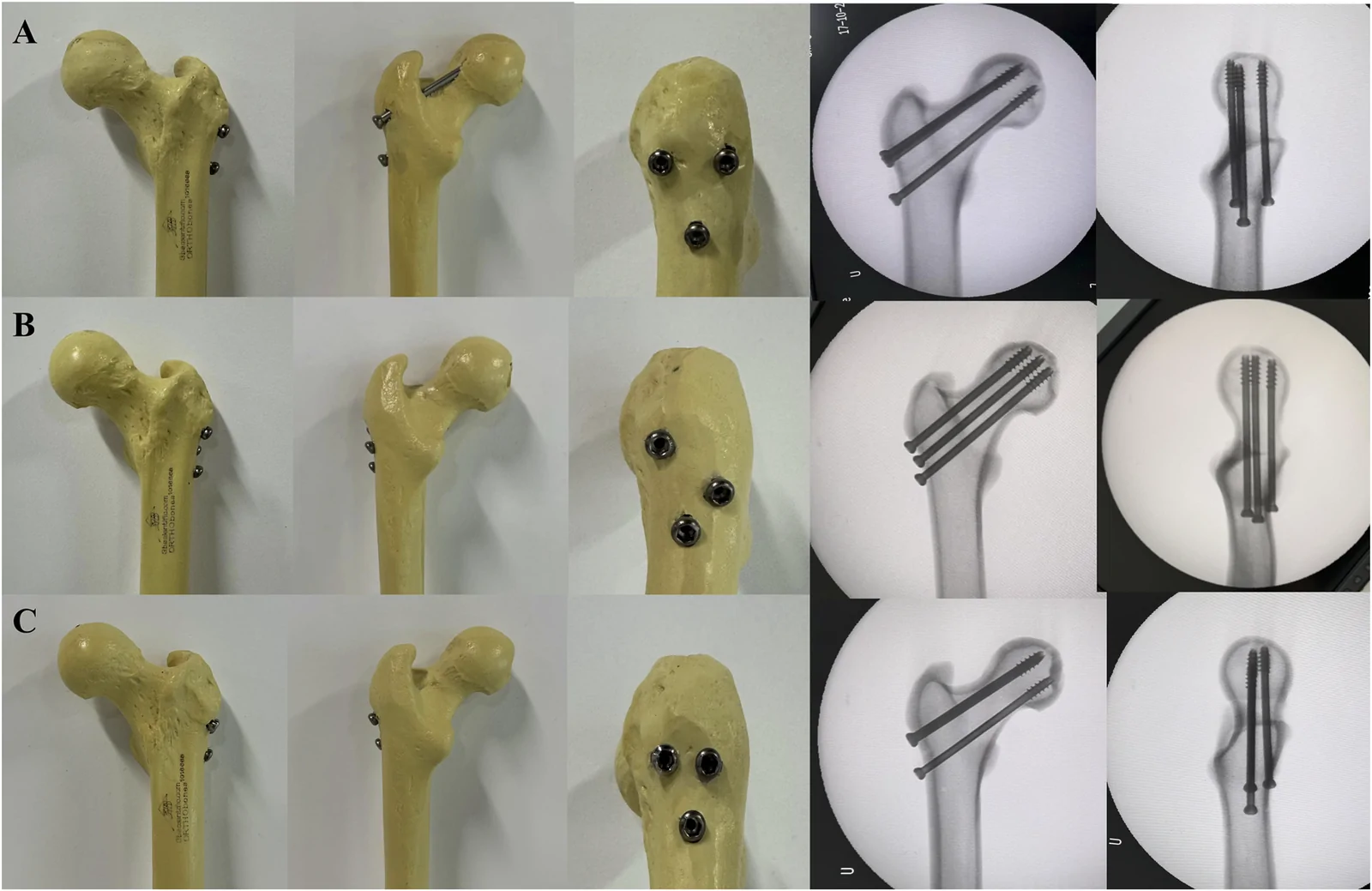
Artificial femoral models for biomechanical testing. Orthobones artificial femurs utilizing the three inverted triangular screw configurations with the posterosuperior screw IOI **(A)**, AI-L**(B)**, and AI-N**(C)**. Each group comprised 15 femurs for subsequent biomechanical experiments.

**FIGURE 3:**
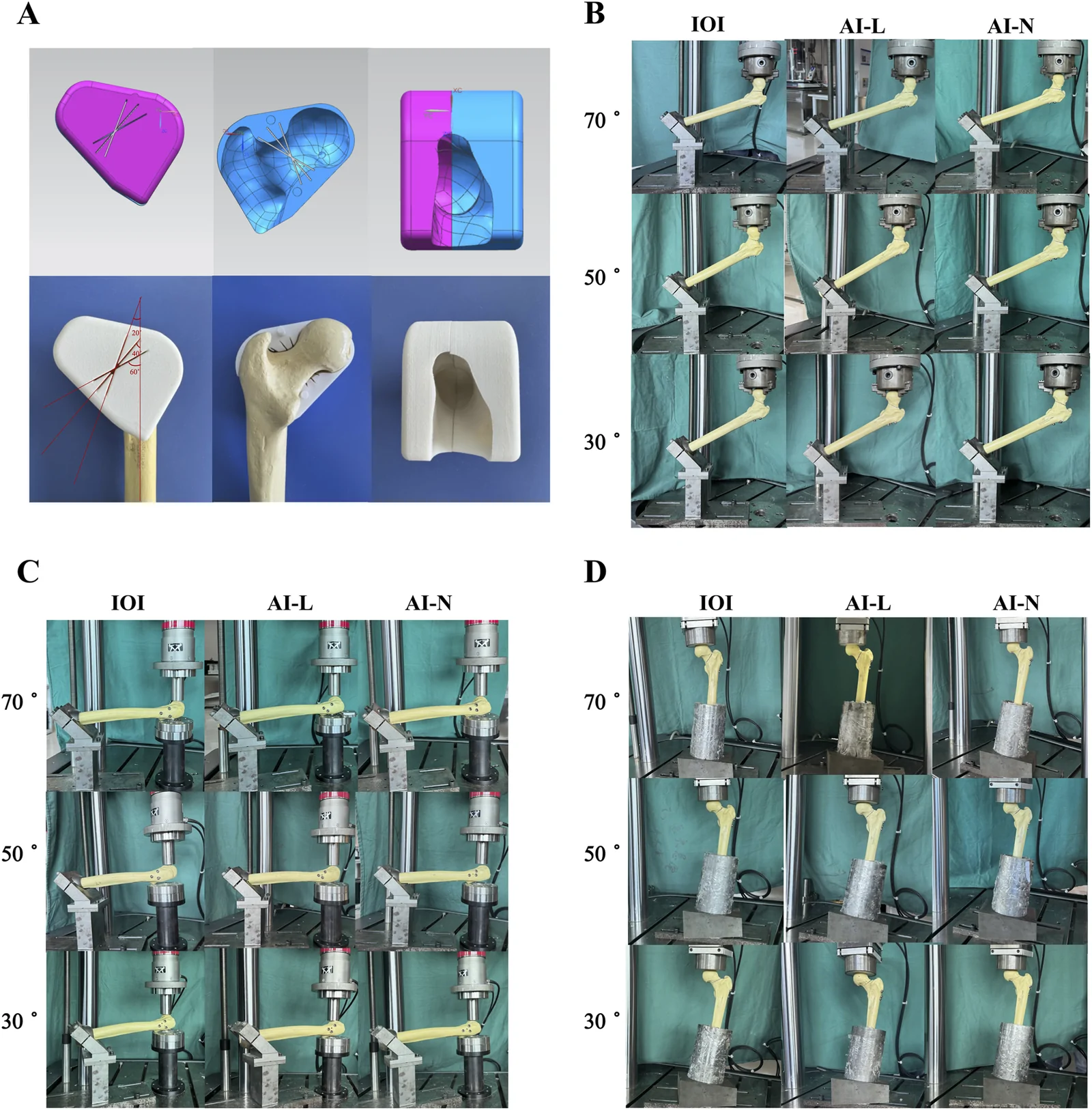
Biomechanical testing setups. **(A)** Custom-made osteotomy jig for creating femoral neck fractures with Pauwels angles of 30°, 50°, and 70°, corresponding to Pauwels type I, II, and III, respectively. **(B)** Torsion test setup: The specimen is immobilized using a custom-made jig to align the femoral neck axis with the torsion axis, and the machine applies torque to the femoral head. **(C)** Femoral position for AP compression testing: The specimen is placed horizontally with a support beam positioned below the lesser trochanter, and the servo-hydraulic material testing system applies loading to the front of the femoral head. **(D)** Femoral position for axial compression and cyclic loading testing: The specimen is abducted by 10° and inclined posteriorly by 9°, and the servo-hydraulic material testing system applies vertical loading to the weight-bearing area of the femoral head.

Based on previous studies, quasi-static tests at non-destructive loading levels were conducted using a servo-hydraulic material testing system (Instron 10000, USA), including torsion test, AP compression test, and axial compression test, followed by cyclic loading test (Wang et al., 2022).

In the torsion test to evaluate anti-rotational stability, an osteotomy was performed 10 cm above the femoral condyle, and the specimen was secured in an inclined position using a custom fixture to align the femoral neck axis with the axis of torsion (Figure 3B). The testing machine applied torque at a torsional speed of 0.5°/s, and torque values were recorded until the femoral head rotated 10° around the femoral neck axis. AP compression test was performed with the specimen placed horizontally with a support beam under the minor trochanter via a custom-made fixture (Figure 3C). The testing machine applied a quasi-static vertical load to the front of the femoral head at a loading rate of 2 mm/min until reaching 5 mm. For axial compression test, the specimen was abducted 10° and tilted backward by 9° (Figure 3D). The testing machine applied a vertical load to the femoral head at a loading rate of 2 mm/min until reaching 5 mm. For cyclic loading test, the specimen was initially loaded with a quasi-static compressive load ranging from 50 N to 200 N at a rate of 15 N/s, followed by a double-peaked physiological compressive cyclic load at a frequency of 2 Hz for a total of 10000 cycles. The valley load was maintained at a constant 200 N, while the peak load was 700 N per cycle, to simulate the partial weight-bearing condition during the initial postoperative fracture union phase (4–6 weeks) (Bergmann et al., 2001).

Record specimen biomechanical data such as torsional angle, torque, displacement, and load. The linear slope of the load-displacement curve is defined as stiffness. Compare the torque at a torsional angle of 10°, the AP stiffness, the axial stiffness in axial compression test, and the maximum displacement in cyclic loading test among the three groups of specimens.

### 2.3 Clinical research

We conducted a retrospective study on femoral neck fractures patients at the First Affiliated Hospital of Dalian Medical University from January 2020 to December 2023. Inclusion criteria included: (1) Adult patients with unilateral femoral neck fractures (age >18 years). (2) Patients who underwent internal fixation with inverted triangular cannulated screws. (3) Patients who underwent postoperative hip CT scans. (4) Postoperative follow-up period exceeded 6 months (5) Complete clinical data. Exclusion criteria included: (1) Pathological, open, or obsolete femoral neck fractures. (2) Anatomical abnormalities of the proximal femur, such as hip osteoarthritis or femoral head AVN. (3) Simultaneous pelvic acetabular fractures or lower limb fractures. (4) Functional impairment before fracture. (5) Loss to follow-up or incomplete data.

The baseline data of the patients were collected, including age, gender, side of fracture, injury mechanism, fracture classification, and time from injury to surgery. Intraoperative data included reduction method, operation time, and quality of fracture reduction. Fracture reduction quality was evaluated on immediate postoperative anteroposterior and lateral radiographs using the Garden’s alignment index (Wen et al., 2025). Postoperative data included the time from surgery to the last follow-up, the major complications included nonunion and femoral head AVN. AVN was defined as the presence of any of the following radiographic signs on plain radiographs obtained from the time of surgery to the final follow-up: regional osteopenia, mottled density, cystic changes, or subchondral sclerosis (Jia et al., 2026). Nonunion was defined as absence of fracture union within 9 months after surgery, with no progressive union observed on serial radiographs over a consecutive 3-month period (Jia et al., 2026).

According to the presence of the posterosuperior cannulated screw IOI in the postoperative hip CT, the patients were divided into the IOI group and the All In group. The diagnosis of IOI was established based on the following CT criteria: axial CT images showing the screw shaft or threads penetrated beyond the femoral neck cortex and then re-entered the bone (Zhang and Tang, 2022) (Supplementary Figure S1). The baseline, intraoperative, and postoperative data were compared between the two groups. Fracture classification and identification of the posterosuperior screw IOI were initially performed by one attending orthopaedic trauma surgeon and subsequently reviewed and confirmed by a senior orthopaedic trauma consultant with over 20 years of experience. Any disagreements were resolved by consensus.

### 2.4 Statistical analysis

SPSS 21.0 was used for statistical analysis. The Kolmogorov-Smirnov test was applied to assess the normality of continuous data. Continuous data following a normal distribution were expressed as mean ± standard deviation, while non-normally distributed continuous data were presented as median (first quartile, third quartile). For normally distributed continuous data with homogeneity of variance, the two independent samples t-test was used for intergroup comparison. For normally distributed data with unequal variance or non-normally distributed data, the Mann–Whitney U test was used. Categorical data were compared using the chi-square test, or Fisher’s exact test when the expected frequency in any cell was less than 5. Univariate and multivariate logistic regression analysis were used to explore the association of configuration with nountion and ANV. The *P* value < 0.05 was considered statistically significant.

## 3 Results

### 3.1 FEA results

Figure 4A showed that in femoral neck fractures of three different Pauwels classifications, the displacement nephogram distribution in the IOI, AI-L, and AI-N groups all exhibited a trend of gradually decreasing from the weight-bearing area of the femoral head toward the distal femur. Under the same conditions, the peak femoral displacement in the IOI group was lower than that in both the AI-L group and the AI-N group (Supplementary Table S1). Figure 4B showed that in the IOI, AI-L, and AI-N groups, the displacement nephogram distribution of the cannulated screws exhibited a trend of gradually decreasing from the tip to the tail. Under the same conditions, the peak displacement of the cannulated screw in the IOI group was lower than that in both the AI-L group and the AI-N group (Supplementary Table S2). Figure 4C showed the femoral stress nephograms for the IOI, AI-L, and AI-N groups. The stress distribution and peak stress values were similar across the three groups. Stress was mainly distributed along the medial aspect of the proximal femoral shaft, and the peak stress was concentrated on the medial side of the femoral neck fracture site. Figure 4D showed the stress distribution and peak stress values of the cannulated screws were similar across the three groups, and the peak stress was mainly concentrated at the screw shaft near the fracture line. The peak stress values of the femur and screws for each group are shown in Supplementary Table S3 and Supplementary Table S4.

**FIGURE 4:**
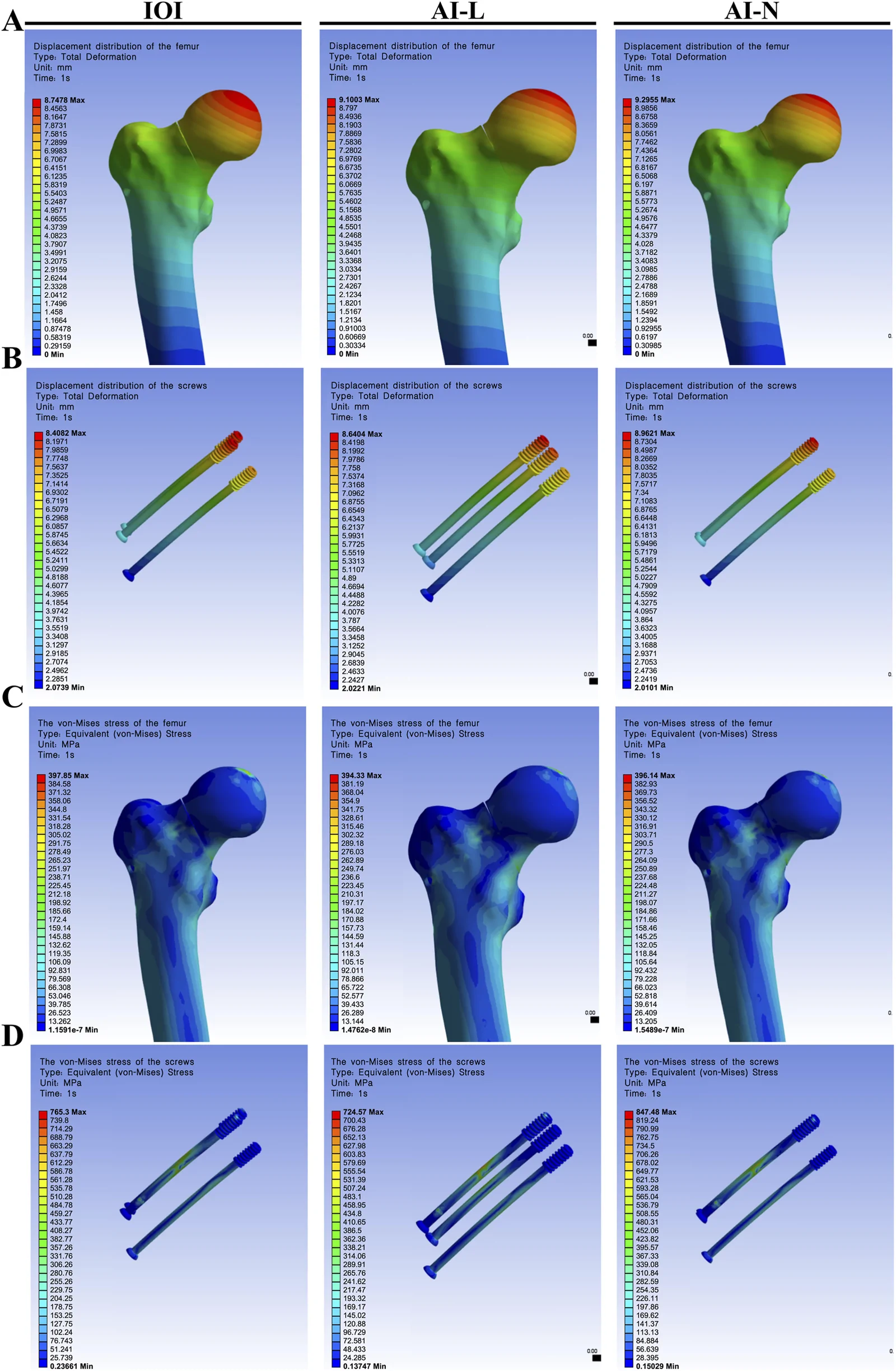
Finite element analysis results. **(A)** Femoral displacement nephograms. **(B)** Cannulated screw displacement nephograms. **(C)** Femoral stress nephograms. **(D)** Cannulated screw stress nephograms.

### 3.2 Biomechanical results

As shown in Figures 5A–C, in the femoral neck fractures of three different Pauwels classifications, the average maximum torque in the IOI group when the femoral head was rotated 10° around the femoral neck axis was greater than that in the AI-N and AI-L groups, with statistically significant difference in Pauwels type II femoral neck fractures. As shown in Figures 5D–I, the IOI group exhibited significantly higher AP stiffness and axial stiffness than the AI-N and AI-L groups across all three Pauwels classifications of femoral neck fractures. In the cyclic loading test, the IOI group exhibited the minimal fracture fragment displacement across all three Pauwels classifications of femoral neck fractures, demonstrating superior biomechanical stability compared to the AI-N and AI-L groups, with statistically significant differences (Figures 5J–L).

**FIGURE 5:**
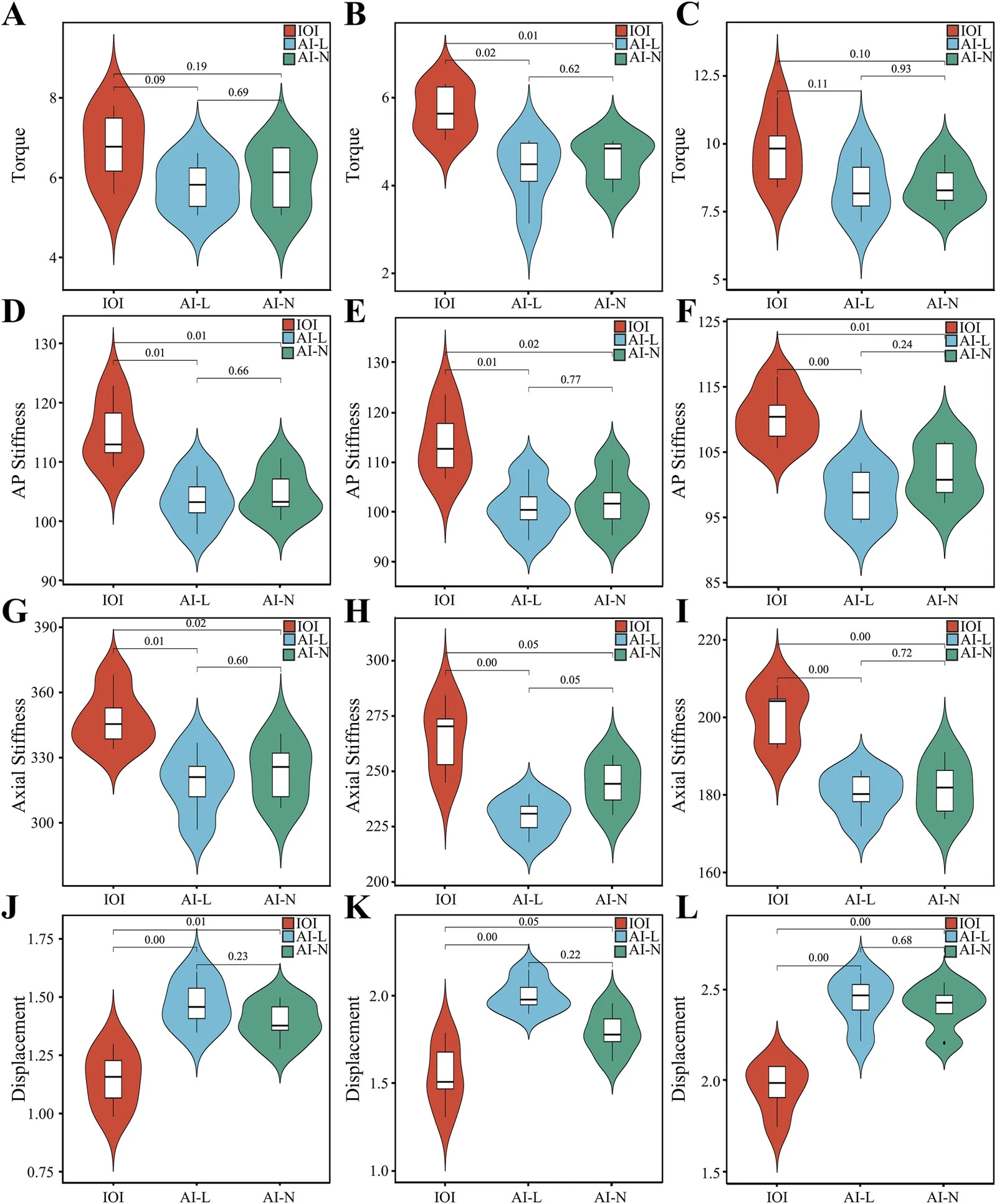
Violin plots comparing the biomechanical performance of the IOI, AI-L, and AI-N configurations in Pauwels type I, II, and III femoral neck fractures. Torque at 10° femoral head rotation in Pauwels type I**(A)**, II**(B)**, and III**(C)** femoral neck fractures; AP stiffness in Pauwels type I**(D)**, II**(E)**, and III**(F)** femoral neck fractures; Axial stiffness in Pauwels type I**(G)**, II**(H)**, and III**(I)** femoral neck fractures; Maximum displacement under cyclic loading in Pauwels type I**(J)**, II**(K)**, and III**(L)** femoral neck fractures.

### 3.3 Clinical results

A total of 91 patients were enrolled in this study, including 32 in the IOI group and 59 in the All In group. The baseline, intraoperative, and postoperative data of the two groups are shown in Table 1. There were no statistically significant differences regarding age, gender, side of fracture, injury mechanism, fracture classification, time from injury to surgery, reduction method, operation time, quality of fracture reduction, and time from surgery to the last follow-up between the two groups. At the last follow-up, the incidence of nonunion in the IOI group and the All In group was 9.38% and 8.47%, respectively, with no statistically significant difference. The incidence of AVN of the femoral head in the IOI group and the All In group at the last follow-up was 15.63% and 15.25%, respectively, with no statistically significant difference. Multivariate logistic regression analysis demonstrated that the incidence of nonunion or AVN did not differ significantly between the IOI and All-In groups (Supplementary Table S5; Supplementary Table S6).

**TABLE 1:** Clinical characteristics and outcomes of the IOI and All In groups.

Variables	IOI	All In	Statistical value	P Value
Age (years)	51.50 (37.50, 56.75)	53.00 (43.00, 59.00)	U = −1.494	0.135
Gender (%)	​	​	χ^2^ = 0.195	0.658
Male	14 (43.75%)	23 (38.98%)	​	​
Female	18 (56.25%)	36 (61.02%)		
Side of fracture (%)	​	​	χ^2^ = 0.325	0.568
Left	15 (46.88%)	24 (40.68%)	​	​
Right	17 (53.13%)	35 (59.32%)		
Injury mechanism (%)	​	​	χ^2^ = 0.030	0.863
Low-energy trauma	25 (78.13%)	47 (79.66%)	​	​
High-energy trauma	7 (21.88%)	12 (20.34%)		
Garden classification	​	​	χ^2^ = 6.030	0.110
I	7 (21.88%)	14 (23.73%)	​	​
II	6 (18.75%)	17 (28.81%)		
III	5 (15.63%)	16 (27.12%)		
IV	14 (43.75%)	12 (20.34%)		
Time to surgery (days)	4.00 (3.00, 6.00)	3.00 (2.00, 6.00)	U = 1.366	0.172
Reduction method	​	​	Fisher’s exact	0.708
Closed reduction	30 (93.75%)	53 (89.83%)	​	​
Open reduction	2 (6.25%)	6 (10.17%)		
Operating duration (minutes)	62.50 (60.00, 90.00)	60.00 (60.00, 75.00)	U = 0.505	0.614
Quality of fracture reduction	​	​	Fisher’s exact	1.000
Level I	29 (90.63%)	54 (91.53%)	​	​
Level II	3 (9.38%)	5 (8.47%)		
Time from surgery to the last follow-up	14.50 (8.25, 24.00)	14.00 (8.00, 24.00)	U = 0.355	0.723
Nonunion	3 (9.38%)	5 (8.47%)	Fisher’s exact	1.000
AVN	5 (15.63%)	9 (15.25%)	Fisher’s exact	1.000

## 4 Discussion

This study systematically evaluated the mechanical performance and clinical outcomes of the posterosuperior screw IOI in the fixation of femoral neck fractures using inverted triangular cannulated screws through finite element analysis, biomechanical experiments, and a retrospective clinical study. Our results show that the IOI configuration exhibits superior performance to the All In configurations in terms of biomechanical properties such as torsional, flexural, shear, and fatigue resistance. However, there was no statistically significant difference between the IOI group and the All In group in the incidence of nonunion and AVN of the femoral head. This finding contradicts the conventional view in the literature that the posterosuperior screw IOI should be avoided as much as possible, as it increases the risk of femoral head perfusion impairment (Yuan et al., 2020).

The mechanical stiffness for cannulated screw fixation of femoral neck fractures is primarily based on the three-point principle (Bout et al., 1997). The tail of the screw is fixed to the lateral cortex of the proximal femur, the shaft adjacent to the femoral neck cortex, and the tip located in the subchondral bone of the femoral head, thus forming a “three-point support”. Previous literature has suggested that disruption of the cortical integrity of the femoral neck compromises mechanical stability. Our study found that the IOI posterosuperior screw penetrates through the cancellous bone of the femoral neck and then inserts into the cortical bone again. In addition to the original “three-point support”, it provides an extra region of cortical support, resulting in “four-point support” with multiple cortical layers. The additional support points distribute the stress more extensively when the screw encounters axial shear and torsional loads, resulting in lower peak stresses. In addition, the cross-sectional area formed by the three screws is larger in the IOI configuration than in the All In configuration. The larger cross-sectional area is associated with higher overall stiffness of the screw configuration (Zhang et al., 2022).

The most controversial and noteworthy finding of our study is that the IOI posterosuperior screw does not increase the risk of nonunion or AVN following femoral neck fractures. Regarding this finding, we propose the following hypotheses: (1) Wu et al. identified the risk zone for damage to the nutrient foramina caused by the IOI posterosuperior screw via CT scanning, and the results showed that not all the IOI posterosuperior screw damaged the nutrient foramen (Wu et al., 2023). Therefore, our findings may be attributable to the IOI posterosuperior screw emerging through the cortical bone of the femoral neck without damaging the superior retinacular artery. (2) Even though the IOI posterosuperior screw may have damaged the superior retinacular artery, the elasticity of the vessel prevented complete occlusion of blood flow. Subsequently, compensatory blood supply of femoral head was provided by anastomotic branches via the microcirculation of the intra-articular arterial network of the femoral head (Sevitt and Thompson, 1965). (3) The superior retinacular artery is embedded in loose connective tissue, with both inner and outer layers consisting of dense fibrous layers, forming a “sandwich-like” structure known as the retinacula of Weitbrecht. This “sandwich-like” structure offers high strength, tensile fracture resistance, and impact resistance, protecting the superior retinacular artery and preventing damage to the vessel caused by the IOI posterosuperior screw (Dou et al., 2018). (4) The initial traumatic injury may have already caused rupture or twisting of the superior retinacular artery at the moment of the femoral neck fracture (Zhao et al., 2017). Under such circumstances, the additional biological risk associated with the IOI posterosuperior screw to the blood supply of the femoral head is negligible.

Based on our study, we recommend reconsidering the clinical value of the IOI posterosuperior screw. Internal fixation of fractures requires a balance between biological protection and mechanical stability, the IOI posterosuperior screw offers marked advantages in mechanical performance, and no additional biological complications such as AVN of the femoral head or nonunion have been observed. This suggests that it may not be appropriate to regard the IOI posterosuperior screw as a surgical technical mistake. Taking into account the hazards of fluoroscopic radiation to both medical staff and patients, frequent intraoperative fluoroscopy to repeatedly adjust the position of the screw should be avoided (Fujibayashi et al., 2021).

However, there are still some limitations in our study. First, the finite element and biomechanical experimental protocols employed in our study could not fully simulate the complex force distribution in the hip joint of patients following femoral neck fracture surgery. Second, the use of synthetic femurs cannot fully replicate the material heterogeneity of human cadaveric bone, and the sample size of five specimens per subgroup is relatively small, which may limit generalizability of the biomechanical findings. Third, our clinical study was a retrospective study with a relatively limited sample size. The proportion of Garden Type IV fractures was higher in the IOI group, which may have confounded comparisons of nonunion and AVN. Furthermore, our outcome assessment was limited to radiographic outcomes, and we did not record functional scores such as femoral neck shortening, internal fixation failure, reoperation rates, or the Harris Hip Score. Importantly, the average follow-up period of approximately 14 months may not be sufficient for a definitive assessment of avascular necrosis, as the incidence of AVN does not stabilize until 2 years. Therefore, our clinical findings should be interpreted with caution and require validation in prospective cohorts with larger sample sizes, longer follow-up periods, and more comprehensive outcome measures.

## 5 Conclusion

This study systematically evaluated the mechanical performance and clinical outcomes of the IOI posterosuperior screw in the inverted triangular cannulated screws fixation of femoral neck fractures through finite element analysis, biomechanical experiments, and a retrospective clinical study. The results indicate that the IOI configuration exhibits superior biomechanical performance compared to the All In configuration, with no statistically significant difference in the incidence of nonunion or AVN of the femoral head. Based on these findings, we suggest that the clinical relevance of the IOI configuration may merit reconsideration, and it may not necessarily need to be strictly avoided as a technical error. However, as this study is retrospective with limited sample size and did not directly assess femoral head blood supply, these findings should be interpreted with caution and require further validation through larger prospective studies with longer follow-up and more comprehensive outcome measures.

## Data Availability

The original contributions presented in the study are included in the article/Supplementary Material, further inquiries can be directed to the corresponding authors.
