# Supplementary material for: Biomechanical and clinical evaluation of the posterosuperior screw in-out-in in the inverted triangular cannulated screws fixation of femoral neck fractures
Source: Front Bioeng Biotechnol. 2026 Sep 23;14:1914058. doi: 10.3389/fbioe.2026.1914058 (PMC13642659; doi:10.3389/fbioe.2026.1914058)
Supplement: Supplementary file 1 [file DataSheet1.docx]

**Supplementary Table 1.** Maximum displacement of the femur models in the IOI, AI-L and AI-N groups in the FEA.

| **Maximum displacement (mm)** | **IOI** |  | **AI-L** |  | **AI-N** |
| --- | --- | --- | --- | --- | --- |
| Pauwels type Ⅰ | 7.09 |  | 7.57 |  | 7.46 |
| Pauwels type Ⅱ | 8.18 |  | 8.57 |  | 8.53 |
| Pauwels type Ⅲ | 8.75 |  | 9.10 |  | 9.30 |

**Supplementary Table 2.** Maximum displacement of the screw models in the IOI, AI-L and AI-N groups in the FEA.

| **Maximum displacement (mm)** | **IOI** |  | **AI-L** |  | **AI-N** |
| --- | --- | --- | --- | --- | --- |
| Pauwels type Ⅰ | 6.80 |  | 7.07 |  | 7.05 |
| Pauwels type Ⅱ | 7.86 |  | 8.13 |  | 8.18 |
| Pauwels type Ⅲ | 8.41 |  | 8.64 |  | 8.96 |

**Supplementary Table 3.** The peak stress of the femur models in the IOI, AI-L and AI-N groups in the FEA.

| **Peak stress (MPa)** | **IOI** |  | **AI-L** |  | **AI-N** |
| --- | --- | --- | --- | --- | --- |
| Pauwels type Ⅰ | 351.62 |  | 350.09 |  | 350.21 |
| Pauwels type Ⅱ | 394.78 |  | 415.52 |  | 405.34 |
| Pauwels type Ⅲ | 397.85 |  | 394.33 |  | 396.14 |

**Supplementary Table 4.** The peak stress of the screw models in the IOI, AI-L and AI-N groups in the FEA.

| **Peak stress (MPa)** | **IOI** |  | **AI-L** |  | **AI-N** |
| --- | --- | --- | --- | --- | --- |
| Pauwels type Ⅰ | 546.85 |  | 413.33 |  | 450.82 |
| Pauwels type Ⅱ | 650.22 |  | 698.18 |  | 682.43 |
| Pauwels type Ⅲ | 765.30 |  | 724.57 |  | 847.48 |

**Supplementary Table 5. Univariate and multivariate logistic regression analysis for incidence of nonunion**

| Covariate | Univariate | | Multivariate | | | | |  |
| --- | --- | --- | --- | --- | --- | --- | --- | --- |
|  | OR (95% CI) | *P* value | OR (95% CI) | | | | *P* value |  |
| IOI/All In | 0.895 (0.200, 4.015) | 0.885 | | 0.987 (0.144, 6.776) | | 0.989 | |  |
| Age (years) | 0.953 (0.899, 1.011) | 0.107 | | 1.077 (0.950, 1.221) | | 0.246 | |  |
| Gender (%) | 0.187 (0.022, 1.585) | 0.124 | | 0.114 (0.006, 2.263) | 0.155 | | | |
| Injury mechanism (%) | 41.417 (4.669, 367.399) | **0.001** | | 48.488 (2.014, 1167.562) | **0.017** | | | |
| Garden classification | 5.074 (1.361, 18.914) | **0.016** | | 3.885 (0.768, 19.666) | 0.101 | | | |
| Side of fracture (%) | 0.729 (0.171, 3.117) | 0.670 | |  |  | | | |
| Time to surgery (days) | 1.121 (0.925, 1.358) | 0.244 | |  |  | | | |
| Reduction method | 4.278 (0.705, 25.959) | 0.114 | |  |  | | | |
| Operating duration (minutes) | 1.008 (0.980, 1.037) | 0.574 | |  |  | | | |
| Quality of fracture reduction | 4.278 (0.705, 25.959) | 0.114 | |  |  | | | |
| Time from surgery to the last follow-up | 0.856 (0.724, 1.013) | 0.071 | |  |  | | | |

**Supplementary Table 6. Univariate and multivariate logistic regression analysis for incidence of AVN.**

| Covariate | Univariate | | Multivariate | |
| --- | --- | --- | --- | --- |
|  | OR (95% CI) | *P* value | OR (95% CI) | *P* value |
| IOI/All In | 0.972 (0.296, 3.193) | 0.963 | 1.831 (0.360, 9.324) | 0.466 |
| Age (years) | 1.044 (0.986, 1.106) | 0.142 | 1.042 (0.965, 1.126) | 0.289 |
| Gender (%) | 1.567 (0.499, 4.915) | 0.442 | 1.929 (0.418, 8.895) | 0.400 |
| Garden classification | 3.009 (1.453, 6.230) | **0.003** | 5.730 (1.928, 17.030) | **0.002** |
| Time from surgery to the last follow-up | 1.057 (1.018, 1.097) | **0.003** | 1.080 (1.024, 1.140) | **0.005** |
| Injury mechanism (%) | 1.040 (0.259, 4.176) | 0.956 |  |  |
| Side of fracture (%) | 2.083 (0.601, 7.223) | 0.247 |  |  |
| Time to surgery (days) | 0.963 (0.784, 1.183) | 0.721 |  |  |
| Reduction method | 1.972 (0.356, 10.940) | 0.437 |  |  |
| Operating duration (minutes) | 1.007 (0.985, 1.030) | 0.527 |  |  |
| Quality of fracture reduction | 1.972 (0.356, 10.940) | 0.437 |  |  |

**Supplementary Figure 1.** The posterosuperior screw was defined as IOI when the screw shaft or threads penetrated beyond the femoral neck cortex and then re-entered the bone on postoperative CT.
